# Supplementary material for: Comparative Analysis of Genetic Alterations, HPV-Status, and PD-L1 Expression in Neuroendocrine Carcinomas of the Cervix
Source: Cancers (Basel). 2021 Mar 10;13(6):1215. doi: 10.3390/cancers13061215 (PMC8001835; doi:10.3390/cancers13061215)
Supplement: Supplementary file 1 [file cancers-13-01215-s001.zip › Supplymentary files/Supplymental figure_S1.pdf]

(A)

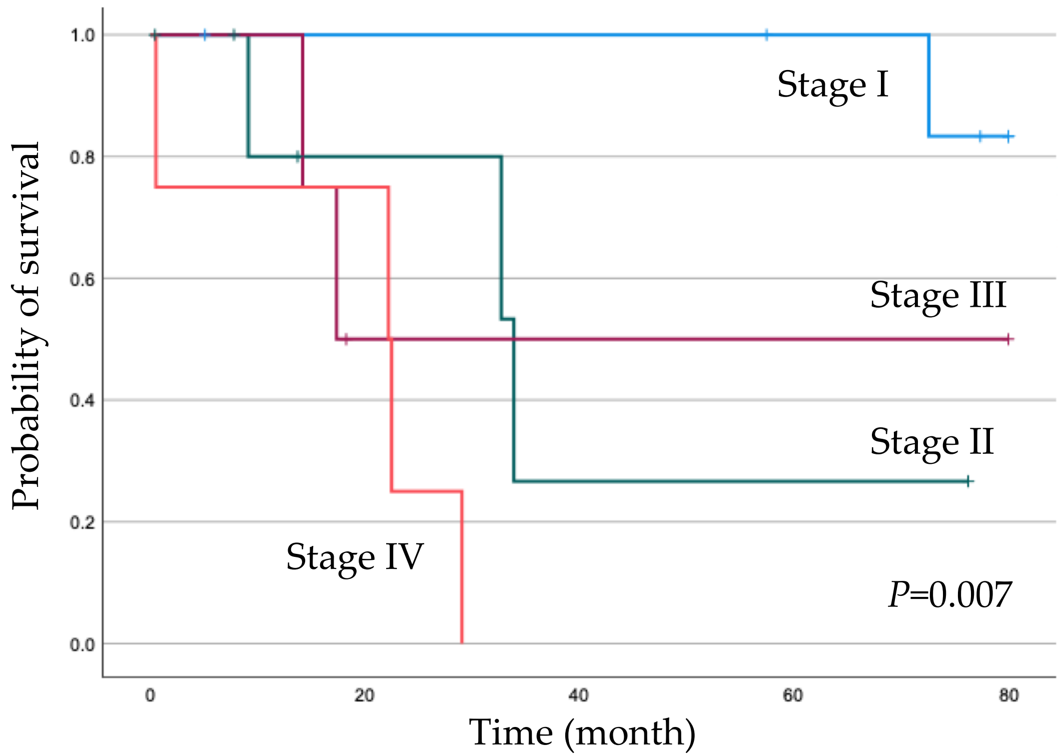

No. at risk

Stage I	8	7	7	5	3
Stage II	7	3	1	0	0
Stage III	4	1	0	0	0
Stage IV	4	3	0	0	0

(B)

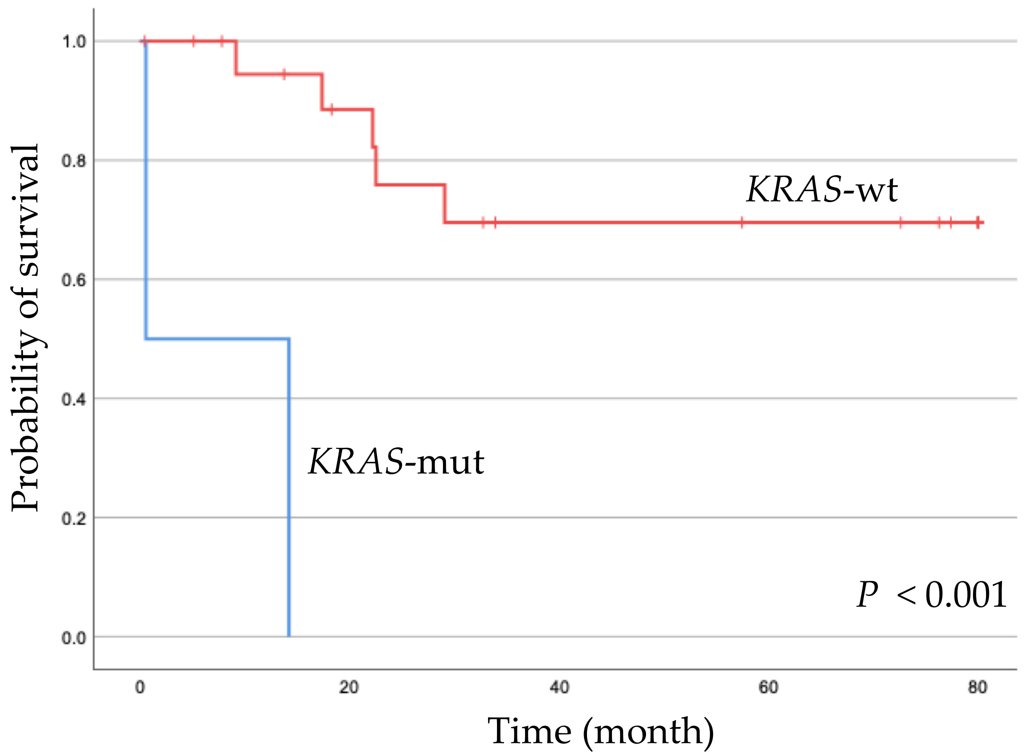

No. at risk

KRAS-mut	2	0	0	0	0
KARS-wt	21	13	8	7	4

Figure S1. Kaplan-Meier analysis of overall survival (OS) for neuroendocrine carcinoma of the cervix (A) OS according to FIGO Stages. OS was significantly decreased among patients with advanced stages (log-rank $p = 0.007$). (B) OS according to *KRAS* gene mutations. Patients with *KRAS* mutations (mut) had significantly shorter OS than those with *KRAS*-wild types (wt) (log-rank $p < 0.001$).
